# The Interplay Between Periodontitis, Caries and Dental Restorations in an Adult Population

**DOI:** 10.1111/jcpe.70043

**Published:** 2025-10-23

**Authors:** Vinay Pitchika, Joachim Krois, Rainer A. Jordan, Søren Jepsen, Falk Schwendicke

**Affiliations:** ^1^ Department of Conservative Dentistry, Periodontology and Digital Dentistry LMU University Hospital, LMU Munich Munich Germany; ^2^ Department of Oral Diagnostics, Digital Health and Health Services Research, Charité – Universitätsmedizin Berlin Berlin Germany; ^3^ Institute of German Dentists (Institut für Deutsche Zahnärzte, IDZ) Cologne Germany; ^4^ Deptartment of Periodontology, Operative and Preventive Dentistry University Hospital of Bonn Bonn Germany

**Keywords:** association, cross‐sectional study, epidemiology, periodontal, risk factors, spatial

## Abstract

**Aim:**

To explore the local co‐occurrence patterns between caries and periodontal parameters (probing depth [PD] and clinical attachment loss [CAL]) and assess whether restorations/caries are spatially associated with poorer periodontal outcomes in a German adult population.

**Materials and Methods:**

Cross‐sectional data from the German Oral Health Study (DMS V, 2014), consisting of 966 adults (35–44 years old) and 1042 seniors (65–75 years old), were analysed. Periodontal examinations were conducted on 12 index teeth (three surfaces), while caries status was evaluated across all tooth surfaces. The relationship between periodontal health and caries was analysed using hierarchical regression models.

**Results:**

Strong associations were found between filled surfaces and increased PD and CAL at both tooth and surface levels. In seniors, crowns and fillings were associated with higher PD (*β* = 0.330–0.372) and CAL (*β* = 0.207–0.368). Surface‐level analyses also showed that early caries lesions were correlated with worse periodontal outcomes (PD: *β* = 0.224–0.591; CAL: *β* = 0.167–0.744).

**Conclusion:**

The findings suggest that early caries lesions and restorations may be linked to poorer periodontal health. These associations were consistent across different surfaces and age groups, highlighting a potential spatial link between caries and periodontal disease.

## 1 Introduction

Periodontitis is a chronic inflammatory disease that affects the supporting structures of teeth, such as the gingiva, periodontal ligament and bone. Dental caries, on the other hand, is a multifactorial disease caused by the demineralisation of tooth structure by acids produced by bacteria in dental plaque. Together, these are among the most common oral diseases, with a global prevalence of approximately 48% (WHO 2022). Both diseases are major contributors to tooth loss and pose a significant oral health burden, even when treated, due to their chronic and progressive nature (Jepsen et al. 2017; Tonetti et al. 2017).

The relationship between periodontitis and caries has recently become a subject of considerable research interest (Frencken et al. 2017; Jepsen et al. 2017; Tonetti et al. 2017). Periodontal pockets and carious lesions both provide environments conducive to the proliferation of pathogenic bacteria. Additionally, inflammatory mediators produced in response to periodontitis may affect salivary gland function, reducing saliva flow and thereby the buffering capacity, which eventually increases the risk of caries. While these diseases differ in their aetiology, they share common aetiological risk factors, such as poor oral hygiene practices, a sugar‐rich diet and smoking (Beklen et al. 2022), which are well‐documented risk factors for both conditions. Perhaps because of the shared aetiological factors, a significant correlation between periodontitis and caries has been reported (Baima et al. 2023; Stangvaltaite‐Mouhat et al. 2024).

A recently published systematic review involving 18 studies found a positive correlation between periodontitis and dental caries, with a pooled odds ratio of 1.57 (95% CI: 1.32–1.87) (Li et al. 2024). The authors also found that the odds of having caries increased by periodontal disease severity (moderate: 1.38 [1.15–1.66]; severe: 2.14 [1.74–2.64]). Similarly, patients with caries were found to have an elevated risk of having periodontal disease (1.79 [1.36–2.35]). Another systematic review assessing the data from 21 cohorts involving 135,018 participants also found similar results (Romandini et al. 2024).

Although numerous studies have assessed the correlation between periodontitis and caries, almost all have done so at the patient level (AlQobaly and Sabbah 2020; Baima et al. 2023; Beklen et al. 2022; Durand et al. 2019; Martínez et al. 2021; Strauss et al. 2019; Tsai et al. 2022). Although this approach provides valuable epidemiological information, it overlooks the localised nature of both diseases and typically assesses the average surface‐level values per patient. For instance, a patient might have deep periodontal pockets of 10 mm on only a few sites, while the remaining sites are periodontally healthy. When averaged across the mouth by calculating the mean probing depth (PD) or clinical attachment loss (CAL), the patient might end up with a low overall mean PD or CAL. This lack of granularity can potentially obscure the presence of site‐specific disease, leading to an underestimation of the disease burden. Emerging evidence has begun to explore tooth‐ or site‐level clustering of oral diseases (Albandar et al. 1995; Stangvaltaite‐Mouhat et al. 2024). However, these studies either limited their assessment to the tooth level or focused on a different age group (early onset periodontitis). On the flip side, surface‐level correlation can provide a more precise assessment of the relationship and can identify patterns of co‐occurrence and interactions. This approach allows for a better understanding of the local aetiological factors and the disease progression by predicting whether a carious lesion is more likely to develop adjacent to periodontal pockets, suggesting direct microbial links. Therefore, in this study, we aimed to assess the correlation between periodontal parameters such as PD and CAL and caries status at the patient, tooth and surface levels.

## 2 Materials and Methods

### 2.1 Study Population

The Institute of German Dentists (Institut der Deutschen Zahnärzte, IDZ) has been conducting repeated cross‐sectional studies, namely the German Oral Health Study (Deutsche Mundgesundheitsstudie, DMS), since 1989, to assess the oral health status of the German population. Participants were selected using a stratified, multistage random sampling strategy based on municipal registries to ensure national representativeness across age, gender and region. Detailed methodology is described in the DMS V methods publication (Jordan et al. 2014). Data from DMS V (2014) included participants across multiple age groups (12 years, 35–44 years, 65–74 years and 75–100 years). For this study, we included only those aged 35–44 and 65–74 years because these age groups represent the population segments where periodontal disease is most prevalent and clinically relevant. The study sample comprised 966 adults (35–44 years old) and 1042 seniors (65– 75 years old). The DMS V was approved by the North Rhine Medical Association Ethics Committee based in Düsseldorf (registration number 2013384). The study is registered in the Health Services Research Database of the German Network for Health Services Research (registration number VfD_DMSV_13_002152).

### 2.2 Dental Examination

Detailed information about the calibration process, interviews, caries and periodontal examination is available in Supporting Information and in a previous publication (Jordan et al. 2014).

### 2.3 Statistical Analyses

Detailed information on the statistical analyses performed in this study is described in Supporting Information.

## 3 Results

Our study population had a mean age of 39.8 ± 3.0 years (adults) and 69.4 ± 3.0 years (seniors), with a relatively equal distribution of males and females (Table 1). Around 11.2% of seniors and 0.4% of adults were edentulous and were excluded from the analyses. Around 70% of the participants were from the West German states, and more adults (42.84%) had higher education than seniors (25.84%). On the contrary, seniors had a higher proportion of non‐smokers (52.56% vs. 46.83%) and a lower proportion of current smokers (11.86% vs. 28.45%) compared to adults. With respect to oral hygiene, more adults used powered toothbrushes and interdental cleaning aids than seniors, and a majority of both adults and seniors reported brushing their teeth at least twice a day. Using the CDC/AAP criteria, the prevalence of moderate or severe periodontitis was 51.6% among adults and 64.7% among seniors. The prevalence of dental caries, defined as (decayed, missing and filled teeth) DMFT > 0, was 97.5% in adults (mean DMFT: 10.08 ± 4.69) and 99.9% in seniors (mean DMFT: 14.88 ± 7.13), indicating a high burden of caries across both age groups. Adults and seniors had approximately 1.93 ± 3.03 and 5.53 ± 4.92 crowns, respectively. While our study sample had a mean PD of 2.32 ± 0.61 and 2.76 ± 0.83 in adults and seniors, respectively, the mean CAL was 2.55 ± 0.83 and 3.75 ± 1.60 in adults and seniors, respectively (Table 2).

**TABLE 1:** Patient characteristics presented as mean ± SD for continuous variables and *N* (%) for categorical variables, stratified by age group.

Variable	Group	Adults (35–44 years)	Seniors (65–75 years)
*N*	—	966	1042
Age	—	39.84 ± 2.95	69.40 ± 2.99
Sex	Male	453 (46.89)	490 (47.02)
	Female	513 (53.11)	552 (52.98)
Region	West German states	683 (70.70)	713 (68.43)
	East German states	283 (29.30)	329 (31.57)
Education	< 10 years	160 (16.60)	480 (47.71)
	10 years	391 (40.56)	266 (26.44)
	> 10 years	413 (42.84)	260 (25.84)
Smoking	Non‐smoker	451 (46.83)	545 (52.56)
	Former smoker	238 (24.71)	369 (35.58)
	Current smoker	274 (28.45)	123 (11.86)
Diabetes	Non‐diabetic	947 (98.03)	873 (83.78)
	Diabetic	19 (1.97)	169 (16.22)
Powered toothbrush use	No	505 (52.28)	694 (66.60)
	Yes	461 (47.72)	348 (33.40)
Interdental cleaning aids	No	367 (37.99)	524 (50.29)
	Floss	338 (34.99)	121 (11.61)
	Dental sticks/picks	48 (4.97)	64 (6.14)
	Interdental brush	65 (6.73)	188 (18.04)
	Multi‐user	148 (15.32)	145 (13.92)
Toothbrushing frequency	≥ 2 times/day	800 (83.07)	874 (84.20)
	< 2 times/day	163 (16.93)	164 (15.80)
Periodontal treatment	No	819 (85.67)	774 (75.44)
	Yes	137 (14.33)	252 (24.56)
Professional oral prophylaxis, last 5 years	≤ 4 times	755 (78.16)	789 (75.72)
	≥ 5 times	211 (21.84)	253 (24.28)

**TABLE 2:** Distribution of the periodontal and caries marker variables in the study sample, stratified by age group.

Variable	Partial mouth examination		Full mouth examination	
	Adults (35–44 years)	Seniors (65–75 years)	Adults (35–44 years)	Seniors (65–75 years)
*N* (%)	885 (91.61)	924 (88.68)	81 (8.39)	118 (11.32)
Mean PD	2.32 ± 0.61	2.76 ± 0.83	2.21 ± 0.47	2.69 ± 1.01
Mean CAL	2.55 ± 0.83	3.75 ± 1.60	2.24 ± 0.50	3.65 ± 1.89
PD ≥ 4 mm	3.58 ± 5.40	4.58 ± 5.63	n/a	n/a
PD ≥ 6 mm	0.31 ± 1.38	0.60 ± 1.75	n/a	n/a
Healthy teeth	—	—	24.09 ± 4.76	11.61 ± 8.33
DT	—	—	0.46 ± 1.34	0.32 ± 1.21
MT	—	—	1.89 ± 3.00	10.72 ± 8.87
FT	—	—	7.73 ± 4.16	3.84 ± 3.92
DFT	—	—	8.18 ± 4.23	4.16 ± 4.11
DMFT	—	—	10.08 ± 4.69	14.88 ± 7.13
Crowns	—	—	1.93 ± 3.03	5.53 ± 4.92
Healthy surfaces	—	—	91.83 ± 23.75	42.66 ± 32.07
DS	—	—	0.86 ± 3.39	0.60 ± 2.83
MS	—	—	9.25 ± 14.33	50.55 ± 40.38
FS	—	—	15.36 ± 9.99	7.95 ± 8.83
DFS	—	—	16.22 ± 10.39	8.55 ± 9.29
DMFS	—	—	25.47 ± 16.61	59.10 ± 36.02

*Note*: Continuous variables and categorical variables were summarised as mean ± standard deviation and number (percentages), respectively.

Abbreviations: CAL, clinical attachment loss; DFT/S, a combination of decayed and filled teeth/surfaces; DMFT/S, a combination of decayed, missing and filled teeth/surfaces; DT/S, decayed teeth/surfaces; FT/S, filled teeth/surfaces; MT/S, missing teeth/surfaces; PD ≥ 4 mm, mean number of sites with probing depth greater than or equal to 4 mm; PD ≥ 6 mm, mean number of sites with probing depth greater than or equal to 6 mm; PD, probing depth.

### 3.1 Patient‐Level Analysis

Mean periodontal PD and CAL values were significantly associated with caries status (Table 3). Although the *β* estimates were of lower magnitude at the patient level, mean CAL was significantly associated with DMFT and had higher *β* estimates compared to the presence of crowns in both adults (*β* = 0.169 [95% CI: 0.129–0.209]) and seniors (0.141 [0.058–0.224]).

**TABLE 3:** Patient‐level analyses: Adjusted estimates (*β*) and their corresponding 95% confidence intervals obtained by regressing mean PD, mean CAL, percentage of sites with PD ≥ 4 mm and percentage of sites with PD ≥ 6 mm from partial‐mouth examination on caries status (teeth indices).

Outcome	Adults (35–44 years)				Seniors (65–75 years)			
	Mean PD, *β* (95% CI)	Mean CAL, *β* (95% CI)	% PD ≥ 4 mm, *β* (95% CI)	% PD ≥ 6 mm, *β* (95% CI)	Mean PD, *β* (95% CI)	Mean CAL, *β* (95% CI)	% PD ≥ 4 mm, *β* (95% CI)	% PD ≥ 6 mm, *β* (95% CI)
Teeth‐based indices								
Healthy teeth	**−0.015** **(−0.024 to −0.006)**	**−0.028** **(−0.040 to −0.016)**	**−0.396** **(−0.642 to −0.149)**	−0.070 (−0.140 to 0.001)	−0.004 (−0.011 to 0.004)	−0.008 (−0.024 to 0.009)	−0.027 (−0.264 to 0.210)	0.003 (−0.082 to 0.088)
DT	**0.106** **(0.075 to 0.136)**	**0.169** **(0.129 to 0.209)**	**2.865** **(2.027 to 3.702)**	**0.649** **(−0.054 to 0.152)**	**0.091** **(0.048 to 0.134)**	**0.141** **(0.058 to 0.224)**	**3.390** **(2.107 to 4.673)**	**0.709** **(0.244 to 1.175)**
MT	**0.028** **(0.012 to 0.044)**	**0.074** **(0.052 to 0.095)**	**0.912** **(0.465 to 1.360)**	0.103 (−0.026 to 0.232)	0.008 (−0.001 to 0.018)	**0.066** **(0.046 to 0.087)**	0.233 (−0.059 to 0.524)	0.084 (−0.021 to 0.188)
FT	0.005 (−0.004 to 0.014)	−0.004 (−0.016 to 0.009)	0.077 (−0.183 to 0.337)	0.019 (−0.056 to 0.093)	0.00 (−0.012 to 0.018)	0.010 (−0.020 to 0.040)	0.171 (−0.287 to 0.628)	0.073 (−0.091 to 0.237)
DFT	**0.014** **(0.005 to 0.023)**	0.011 (−0.001 to 0.023)	**0.320** **(0.068 to 0.572)**	**0.074** **(0.002 to 0.146)**	0.012 (−0.002 to 0.027)	0.025 (−0.003 to 0.054)	**0.525** **(0.094 to 0.957)**	0.143 (−0.012 to 0.298)
DMFT	**0.021** **(0.012 to 0.019)**	**0.031 (0.020 to 0.042)**	**0.549** **(0.311 to 0.787)**	**0.096** **(0.028 to 0.164)**	**0.019** **(0.007 to 0.030)**	**0.096** **(0.074 to 0.118)**	**0.626** **(0.293 to 0.959)**	**0.197** **(0.078 to 0.317)**
Crowns	**0.014** **(0.001 to 0.027)**	0.014 (−0.004 to 0.031)	0.270 (−0.091 to 0.631)	0.087 (−0.016 to 0.190)	−0.004 (−0.017 to 0.008)	**−0.074** **(−0.098 to −0.049)**	**−0.279** **(−0.651 to 0.094)**	−0.133 (−0.267 to 0.000)

*Note*: Adjusted for age, sex, region (east/west), education, smoking, diabetes status, toothbrushing frequency, powered toothbrush use, interdental cleaning aids use, periodontal treatment and regular professional oral prophylaxis. Bold numbers indicate statistically significant associations.

Abbreviations: % PD ≥ 4 mm, percentage of sites with probing depth greater than or equal to 4 mm; %PD ≥ 6 mm, percentage of sites with probing depth greater than or equal to 6 mm; CAL, clinical attachment loss; DFT, a combination of decayed and filled teeth; DMFT, a combination of decayed, missing and filled teeth; DT, decayed teeth; FT, filled teeth; MT, missing teeth; PD, probing depth.

### 3.2 Tooth‐Level Analysis

At the tooth level, the analysis revealed that teeth D_3‐4_ caries lesions were associated with higher PD (0.558 [0.330–0.785]) and CAL (0.511 [0.259–0.762]) in adults. On the contrary, D_3‐4_ caries lesions were inversely associated with PD (−0.155 [−0.425 to −0.115]) and CAL (−0.522 [−0.890 to −0.154]) in seniors (Table 4). While the presence of fillings was significantly associated with higher PD (0.348 [0.297–0.399]) and CAL (0.288 [0.231–0.344]) in adults, teeth with crowns were associated with higher PD in both adults (0.170 [0.105–0.236]) and seniors (0.225 [0.158–0.283]). Furthermore, the tooth type was found to be a significantly associated factor, with stronger correlation in premolars and molars.

**TABLE 4:** Tooth‐level analysis: Adjusted estimates (*β*) and their corresponding 95% confidence intervals obtained by regressing mean PD and mean CAL from full‐mouth examination on caries status. Mixed‐effects models were constructed in the order of tooth type > jaw > patient ID.

Outcome	Adults (35–44 years)		Seniors (65–75 years)	
	Mean PD, *β* (95% CI)	Mean CAL, *β* (95% CI)	Mean PD, *β* (95% CI)	Mean CAL, *β* (95% CI)
Caries status (healthy surface)	**Ref**.	**Ref**.	**Ref**.	**Ref**.
D_1‐2_ lesions	0.109 (−0.019 to 0.236)	**0.248** **(0.109 to 0.388)**	**0.346** **(0.061 to 0.631)**	0.235 (−0.134 to 0.603)
D_3‐4_ lesions	**0.558** **(0.330 to 0.785)**	**0.511** **(0.259 to 0.762)**	−0.155 (−0.425 to 0.115)	**−0.522** **(−0.890 to −0.154)**
Filling	**0.348** **(0.297 to 0.399)**	**0.288** **(0.231 to 0.344)**	**0.298** **(0.216 to 0.379)**	**0.182** **(0.074 to 0.290)**
Crown	**0.170** **(0.105 to 0.236)**	n/a	**0.225** **(0.158 to 0.283)**	n/a
Tooth type (Incisor)	**Ref**.	**Ref**.	**Ref**.	**Ref**.
Canine	**0.089** **(0.035 to 0.143)**	**0.121** **(0.051 to 0.191)**	**0.092** **(0.009 to 0.175)**	−0.068 (−0.244 to 0.107)
Premolar	**0.265** **(0.216 to 0.314)**	**0.334** **(0.268 to 0.401)**	**0.281** **(0.198 to 0.364)**	0.156 (−0.030 to 0.342)
Molar	**0.586** **(0.536 to 0.636)**	**0.706** **(0.638 to 0.774)**	**0.709** **(0.617 to 0.800)**	**1.200** **(0.987 to 1.412)**

*Note*: Adjusted for tooth type, surface of the tooth, age, sex, region (east/west), education, smoking, diabetes status, toothbrushing frequency, powered toothbrush use, interdental cleaning aids use, periodontal treatment and regular professional oral prophylaxis. Bold numbers indicate statistically significant associations.

Abbreviations: CAL, clinical attachment loss; D_1‐2_ lesions, caries lesions extending until enamel; D_3‐4_ lesions, caries lesions extending until dentine.

### 3.3 Surface‐Level Analysis

At the surface level, D_1‐2_ caries lesions were significantly associated with PD at all surfaces in both adults and seniors; however, D_3‐4_ lesions were associated only with CAL in disto‐lingual mid‐buccal surfaces in adults. Filled surfaces and crowns were significantly associated with PD at mesio‐buccal (fillings: 0.185 [0.147–0.223], crowns: 0.260 [0.206–0.313]), disto‐lingual (fillings: 0.162 [0.120–0.204], crowns: 0.222 [0.162–0.282]) and mid‐buccal (fillings: 0.125 [0.062–0.187], crowns: 0.217 [0.164–0.269]) surfaces in adults. Crowns were significantly associated with CAL at all surfaces (mesio‐buccal: 0.270 [0.220–0.321], disto‐lingual: 0.207 [0.160–0.253], mid‐buccal: 0.192 [0.113–0.271]). Associations of similar magnitude were observed among seniors with fillings or crowns and PD or CAL (Table 5).

**TABLE 5:** Surface‐level analyses: Adjusted estimates (*β*) and their corresponding 95% confidence intervals obtained by regressing mean PD and mean CAL from partial‐mouth examination on caries status. Mixed‐effects models were constructed in the order of tooth type > jaw > patient ID.

Outcome	Adults (35–44 years)		Seniors (65–75 years)	
	Mean PD, *β* (95% CI)	Mean CAL, *β* (95% CI)	Mean PD, *β* (95% CI)	Mean CAL, *β* (95% CI)
Correlating PD and CAL on MB sites with caries status on M surface				
Caries status (healthy surface)	**Ref**.	**Ref**.	**Ref**.	**Ref**.
D_1‐2_ lesions	**0.118** **(0.003 to 0.232)**	−0.028 (−0.176 to 0.121)	**0.318** **(0.111 to 0.524)**	**0.690** **(0.379 to 1.001)**
D_3‐4_ lesions	0.144 (−0.001 to 0.290)	0.172 (−0.019 to 0.363)	0.117 (−0.147 to 0.380)	−0.030 (−0.443 to 0.382)
Filling	**0.185** **(0.147 to 0.223)**	**0.270** **(0.220 to 0.321)**	**0.268** **(0.192 to 0.345)**	**0.368** **(0.248 to 0.487)**
Crown	**0.260** **(0.206 to 0.313)**	n/a	**0.372** **(0.309 to 0.435)**	n/a
Correlating PD and CAL on DL sites with worst caries status on D and L surfaces				
Caries status (healthy surface)	**Ref**.	**Ref**.	**Ref**.	**Ref**.
D_1‐2_ lesions	**0.197** **(0.088 to 0.306)**	**0.173** **(0.053 to 0.292)**	**0.297** **(0.093–0.501)**	**0.344** **(0.024 to 0.663)**
D_3‐4_ lesions	0.111 (−0.047 to 0.268)	**0.182** **(0.010 to 0.355)**	**0.552** **(0.296 to 0.808)**	0.132 (−0.281 to 0.545)
Filling	**0.162** **(0.120–0.204)**	**0.207** **(0.160 to 0.253)**	**0.234** **(0.160 to 0.307)**	**0.207** **(0.091 to 0.323)**
Crown	**0.222** **(0.162–0.282)**	n/a	**0.370** **(0.307 to 0.432)**	n/a
Correlating PD and CAL on B sites with caries status on B surface				
Caries status (healthy surface)	**Ref**.	**Ref**.	**Ref**.	**Ref**.
D_1‐2_ lesions	**0.224** **(0.126 to 0.321)**	**0.167** **(0.044 to 0.290)**	**0.591** **(0.307 to 0.875)**	**0.744** **(0.316 to 1.172)**
D_3‐4_ lesions	0.098 (−0.103 to 0.298)	**−0.256** **(−0.507 to −0.006)**	0.194 (−0.087 to 0.476)	−0.292 (−0.725 to 0.140)
Filling	**0.125** **(0.062 to 0.187)**	**0.192** **(0.113 to 0.271)**	**0.110** **(0.001 to 0.219)**	0.063 (−0.107 to 0.232)
Crown	**0.217** **(0.164 to 0.269)**	n/a	**0.330** **(0.273 to 0.386)**	n/a

*Note*: Adjusted for age, sex, region (east/west), education, smoking, diabetes status, toothbrushing frequency, powered toothbrush use, interdental cleaning aids use, periodontal treatment and regular professional oral prophylaxis. Bold numbers indicate statistically significant associations.

Abbreviations: CAL, clinical attachment loss; D_1‐2_ lesions, caries lesions extending until enamel; D_3‐4_ lesions, caries lesions extending until dentine; PD, probing depth.

## 4 Discussion

Unlike previous research, which primarily focused on patient‐level correlations, the present study delves into the complex relationship between periodontitis and caries by analysing their correlations at the patient, tooth and surface levels. This offers a more granular analysis, which is crucial for understanding the localised interactions between these two prevalent oral diseases.

Our findings reveal significant associations between periodontal parameters and caries status, with the strength of these associations varying depending on the level of analysis. At the patient level, higher mean PD and mean CAL were consistently associated with worse caries status, a result that is in agreement with the broader literature (Baima et al. 2023; Li et al. 2024; Romandini et al. 2024). However, this study extends beyond previous work by demonstrating that the associations persist at the tooth level, and much strongly at the surface levels.

Our study's main strength is the availability of large‐scale cross‐sectional data that is representative of Germany, and associates PD and CAL with caries at the patient, tooth and surface levels. We used a mixed‐effects model that considers tooth type and jaw position, which allowed us to isolate the effects of periodontal health on caries status more effectively, further enhancing the robustness of our findings. However, our study also had a few limitations. First, the cross‐sectional design limited our ability to infer causality between periodontal markers and caries (Altman and Krzywinski 2015). While we can establish correlations, the temporal relationship between these diseases remains unclear. Caries and periodontitis progress at different rates and with different episodicity; that is, periodontal breakdown may occur in bursts, whereas caries tends to progress more steadily. Given the age distribution in our study, it is also likely that caries onset occurred earlier in life, before the development of significant periodontal deterioration. This sequencing could partly shape the associations observed, but our data could not disentangle these temporal dynamics. As a result, we could not determine whether one condition preceded or accelerated the other or whether they shared common risk factors. Second, while we included only those aged 35–44 and 65–74 years in this study, this selection was deliberate because of the higher prevalence of periodontitis and sufficient retention of natural teeth in these groups. Therefore, while our findings are representative of these key risk groups within Germany, they may not be generalisable to younger or much older populations. Third, the partial periodontal recordings (three sites on 12 index teeth) may not capture the full extent of periodontal disease, particularly in cases where disease is localised to non‐examined sites. It is also known to result in considerable bias with unclear direction in estimating the mean PD (Beltrán‐Aguilar et al. 2012) and a dilution of effect estimates towards the null (Akinkugbe et al. 2015). To address these limitations, we limited our tooth‐level analyses to 199 participants who underwent full‐mouth periodontal recordings, thus ensuring a comprehensive assessment on all sites. However, this smaller subsample restricted the statistical power, decreased the generalisability of findings and hindered the creation of a robust calibration model or a formal quantitative bias analysis. Fourth, measurement error from partial recordings may also vary according to the caries status, potentially introducing differential bias into our estimates. Also, in the surface‐level analyses, only four caries surfaces per tooth were included to align with available periodontal probing sites, which may underestimate the overall caries burden. Occlusal surfaces were excluded because of a lack of direct periodontal correspondence and potential misclassification; however, they were considered in tooth‐ and patient‐level analyses. Fifth, Because of inconsistencies in the assessment of root caries in DMS V, this study only assessed coronal caries. To maintain consistency across waves, root caries were excluded from the current study. Sixth, a potential limitation is that tooth loss can result from various causes, including periodontal disease; yet it was included in caries indices (e.g., DMFT, DMFS) in the patient‐level analysis. However, complementary models using indices that exclude missing teeth (DT/DS, FT/FS, DFT/DFS) showed consistent associations. Furthermore, missing teeth were not included in tooth‐ or surface‐level analyses. Seventh, some attachment loss at the site level may be caused by factors other than periodontitis, such as subgingival caries or tissue alterations due to crown preparations or subgingival margins. Because CAL was analysed as an overall periodontal parameter, we could not fully distinguish these non‐periodontal causes of attachment loss, which may have introduced some misclassification bias in the surface‐level analysis. Eighth, our study relied on visual assessments to determine caries status. Although standardised and widely accepted (Gimenez et al. 2015), it may not be as sensitive as radiographic methods for detecting early lesions, particularly in the proximal areas (Schwendicke et al. 2015, 2021). Ninth, although we could adjust for a number of covariates, residual confounding from variables not considered in the models, such as socio‐economic status, diet, medications, physical activity, stress and so on, could modify the associations in the models (Wunsch et al. 2006). Finally, because of the homogeneous nature of the data, our results might not be externally valid for non‐European populations.

Despite having strong associations in our study, some inconsistencies were observed, particularly with respect to the negative associations between the presence of crowns and periodontal parameters in seniors at patient‐level analyses. One possible explanation for this finding could be related to the challenges in accurately performing periodontal measurements, particularly recessions, in older people. Seniors often experience gingival recession due to ageing or previous periodontal disease (West et al. 2024), which can expose the crown margins (Carvalho and Lussi 2017; Ketterl 1983). This could possibly lead to better toothbrushing efficiency and less plaque accumulation at the crown margins compared to younger adults with less gingival recession. Additionally, seniors may have undergone more extensive dental work over time, leading to a heterogeneous mix of restorations—some of which may have been well maintained—or further complicating the relationship between crowns and periodontal parameters at the patient level in this age group.

Such differences in the direction and magnitude of associations between adults and seniors might also reflect age‐related changes such as alterations in saliva composition, immune response or the mechanical properties of the periodontal apparatus. These factors could differentially affect how periodontal disease and caries interact within different age groups. For example, seniors may experience more pronounced periodontal breakdown around certain restorations or in specific areas of the mouth, compared to younger adults. Moreover, differences in dental care habits (Pitchika et al. 2022), access to dental services and the quality of restorations over a lifetime could further contribute to these variations. Seniors who have retained their teeth may represent a survivor cohort with better overall oral hygiene and regular dental care, possibly leading to better periodontal outcomes around restorations compared to adults who are more likely to present with untreated or poorly managed dental conditions. However, we did not observe such negative associations when analysing at the tooth level and surface level, further supporting our scientific approach.

We observed a correlation between periodontal parameters and caries status at the tooth level using full‐mouth periodontal examination from the subset as well. The correlation was much stronger in the premolars or molars compared to the incisors or canines, which could be attributed to improper brushing habits in the posterior region. This might also suggest that the spatial distribution of periodontal disease within the mouth may play a role in caries development (Proctor et al. 2020), possibly due to localised bacterial interactions or changes in the oral environment that favour demineralisation. Although the correlation between mean PD and CAL and D_1‐2_ lesions was not very clear in this subset, a stronger association with D_3‐4_ caries lesions (only in adults), the presence of crowns, followed by fillings, was apparent. An inverse relationship was observed, where seniors with D_3‐4_ caries lesions had lower PD and CAL. Possible explanations include tooth loss bias, where missing teeth are the most affected by caries and periodontitis, leaving healthier teeth behind. Furthermore, larger carious lesions can destroy the tooth structure, particularly in the cervical or proximal areas, affecting the cemento‐enamel junction (CEJ). This damage might lower CAL measurements, as the CEJ, which is used as a reference for measuring CAL, is compromised. Lastly, the long treatment history of seniors might have changed their periodontal environment, affecting the CAL readings. These interpretations are speculative, highlighting the need for longitudinal studies to determine whether this association is biologically significant or a result of treatment dynamics. This inverse relationship highlights the complex interplay between periodontal disease and caries. It could be speculated that the presence of substantial carious lesions might be protective against the deepening of periodontal pockets, contrary to what might be expected in a more typical progression of periodontal disease. This underscores the importance of considering the directionality and context of such associations when interpreting the results, particularly in older populations where these dynamics may differ from younger cohorts. However, because of the small sample size in this subset, the results from our tooth‐level analyses should be interpreted with caution. Our findings are in line with previous efforts to explore localised patterns of co‐occurrence. For instance, Stangvaltaite‐Mouhat et al. (2024) recently demonstrated tooth‐level co‐occurrence between caries and periodontal inflammation in an adult population. Their results, derived from full‐mouth data, support the notion that site‐level factors, such as restorative history or anatomical challenges, may contribute to overlapping disease patterns.

In addition to patient‐level and tooth‐level analyses, our study provided valuable insights into the surface‐level correlations between periodontal parameters and caries status. Our findings indicate that teeth with caries lesions, fillings or crowns presented with deeper PD and greater CAL at the corresponding surfaces. The underlying mechanisms for this association are likely multifactorial. One plausible explanation is a bidirectional relationship, where periodontal disease not only contributes to caries development but may also be influenced by previous caries treatment. Fillings and crowns can sometimes have subgingival margins, which can be challenging to clean and can create plaque‐retentive niches that not only trap cariogenic bacteria but also foster the kind of inflammatory environment that potentially leads to the development of periodontal pockets (Lang et al. 1983). These restorations, as well as untreated caries, can alter the local biofilm balance, supporting bacterial communities that influence both diseases simultaneously (Mira et al. 2017). Another possible pathway is that the restorative materials may not be compatible with some patients, resulting in long‐term irritation to the surrounding gingival tissue (Willershausen et al. 2001). This can lead to pocket formation over time (Isler et al. 2018). Once deep pockets form, they can serve as reservoirs for cariogenic bacteria, or the inflammatory environment may directly contribute to caries progression by affecting the protective functions of saliva or altering local pH levels (Baliga et al. 2013; Koppolu et al. 2022; Lăzureanu et al. 2021). Additionally, restorations that alter the normal occlusal forces on a tooth are believed to contribute to periodontal destruction through increased stress on the periodontal ligament and alveolar bone; however, the evidence is rather weak (Campiño et al. 2019; Fan and Caton 2018; Harrel 2003). The surface‐level data also revealed that the association between periodontitis and caries or restorations was more pronounced on certain surfaces such as the mesio‐buccal and disto‐lingual surfaces than the mid‐buccal surfaces. The fact that these associations were particularly pronounced on posterior and approximal surfaces also hints at an anatomical dimension: these surfaces are more difficult to clean, more likely to accumulate plaque and more frequently restored, which could amplify the link between caries and periodontal disease (Jepsen 1983; Van der Weijden et al. 1998), especially in older adults with compromised dexterity (Pitchika et al. 2021).

While the associations we observed were statistically significant, their clinical implications are equally important. Our findings highlight the importance of considering spatial disease patterns in oral health assessment. The consistent associations between early caries lesions or restorations and deeper periodontal parameters suggest that localised risk areas may exist within the oral cavity. Clinicians should be cautious when evaluating patients with an extensive restorative history, as these sites may warrant closer monitoring for future periodontal breakdown. Therefore, a more holistic approach in everyday dental practice—where one considers caries and periodontal disease together, rather than in isolation−could support more personalised prevention, diagnosis and treatment strategies. Future studies may investigate whether bacterial composition or load differs in patients with a higher restorative burden or at specific surfaces with restorations, which could further explain the observed spatial associations and inform more targeted interventions.

## 5 Conclusion

In conclusion, while periodontitis and dental caries are distinct diseases, they are correlated, and patients with one condition may be at a higher risk of developing the other. Therefore, it is important for dental professionals to be aware of this correlation and to address both conditions simultaneously in their treatment plans.

## Author Contributions

Vinay Pitchika contributed to conception and design, data analysis and interpretation, drafting and critically revising the manuscript. Joachim Krois, Søren Jepsen and Falk Schwendicke contributed to conception and design, interpretation and critically revising the manuscript. Rainer A. Jordan contributed to conception and design, data acquisition and critically revising the manuscript. All authors gave their final approval and agreed to be accountable for all aspects of the work.

## Conflicts of Interest

The authors declare no conflicts of interest.

## Supporting information


**Data S1:** jcpe70043‐sup‐0001‐supinfo.docx.

## Data Availability

The data that support the findings of this study are available on request from the corresponding author. The data are not publicly available due to privacy or ethical restrictions.
