# Supplementary material for: The Interplay Between Periodontitis, Caries and Dental Restorations in an Adult Population
Source: J Clin Periodontol. 2025 Oct 23;53(1):72–81. doi: 10.1111/jcpe.70043 (PMC12695450; doi:10.1111/jcpe.70043)
Supplement: Supplementary file 1 — Data S1: jcpe70043‐sup‐0001‐supinfo.docx. [file JCPE-53-72-s001.docx]

**Supplementary material**

*Dental examination*

Calibrated and licensed dentists performed dental examinations, recording the full-mouth surface-based caries status visually (World Health Organization, 1997). Several variables were considered as coronal caries status markers at the patient level, including the number of caries-free healthy teeth/surfaces, decayed teeth/surfaces (DT/S), missing teeth/surfaces (MT/S, irrespective of the cause of tooth loss), filled teeth/surfaces (FT/S), and the combined indices (DFT/S and DMFT/S), along with the presence of crowns. For the tooth-level analyses, the caries status on the coronal surfaces of the teeth was assessed, with the worst possible outcome being assigned to the teeth. The hierarchy from the best to worst was: caries-free healthy tooth, caries lesions affecting only enamel (D_1-2_), caries lesions affecting dentine (D_3-4_), and surfaces with fillings, followed by crowns. For surface-level analyses, the coronal caries status was determined based on the condition of each corresponding surface.

Periodontal examinations were performed on a partial mouth basis on mesiobuccal, mid-buccal, and distolingual sites from the following index teeth: 11, 16, 17, 24, 26, 27, 31, 36, 37, 44, 46, and 47. Mean PD and CAL were calculated from all sites on the patient and tooth levels. A subset of participants (81 adults and 118 seniors) had full-mouth periodontal examinations that were performed on six sites (mesio-buccal, mid-buccal, disto-buccal, mesio-oral, mid-oral, disto-oral) on 28 teeth, respectively. Detailed information about the calibration process, interviews, caries and periodontal examination can be accessed through a previous publication (Jordan et al., 2014).

*Statistical analyses*

Descriptive statistics and analyses were stratified by age group (adults: 35-44 years, seniors: 65-74 years) (Table 1). Mean PD (dentate only) and mean CAL (dentate only) were considered the periodontal outcome variables, which were regressed on the caries status at the patient, tooth, and surface levels. Partial-mouth periodontal examination data were used for all analytic levels (patient, tooth, and surface). As a part of sensitivity analyses, deepest PD and deepest CAL per tooth were considered as the outcomes for tooth-level analyses and were regressed on the caries status (Suppl. Table 2).

For patient-level analyses, the mean PD and mean CAL per patient were used as outcome variables and regressed on patient-level caries indicators, including the number of healthy teeth (and surfaces), DT/S, MT/S, FT/S, DFT/S, DMFT/S scores, and the number of crowns. For tooth-level analyses, the mean PD and mean CAL per tooth were used as outcome variables and were regressed on the caries status of the corresponding tooth, categorised ordinally as healthy, D_1–2_ lesion, D_3–4_ lesion, filled, or crowned. For the surface-level analyses, due to the presence of periodontal measurements only on three sites, which did not correspond to the 5-surface caries assessment, a surface-matching was performed: i) PD and CAL values on the mesiobuccal sites were correlated with the caries status on the mesial surface of the corresponding tooth; ii) PD and CAL values on the distolingual surfaces were correlated with the worst caries status on the distal and lingual surfaces; iii) PD and CAL on mid-buccal sites were correlated with the caries status on the buccal surface.

Surface- and tooth-level analyses were conducted using linear mixed-effects models. Patient-level analyses used standard linear regression, while tooth- and surface-level analyses accounted for the multi-level data structure (multiple sites nested within teeth, teeth nested within jaws, and jaws nested within patients). For each model, random intercepts were specified for patient ID, jaw, and tooth type to account for within-subject clustering. The covariance structure was specified as unstructured (cov[unstr]), allowing variances and covariances among random effects to be freely estimated. Models were fitted using restricted maximum likelihood estimation. Both outcomes (PD and CAL) were modelled as continuous variables, with predictor variables included linearly after confirming no meaningful non-linear effects.

All models were adjusted for covariates such as age, sex (male vs. female), region (West German states vs. East German states), education (< 10 years, 10 years vs. > 10 years), smoking (non-smoker, former smoker, vs. current smoker), diabetes (non-diabetic vs. diabetic), powered toothbrush use (no vs. yes), interdental cleaning aids used (no, dental floss, dental sticks/picks, interdental brush vs. multi-user), toothbrushing frequency (≥ 2 times/day vs. < 2 times/day), periodontal treatment in the last 5 years (no vs. yes), and having an professional oral prophylaxis in the last 5 years (≤ 4 times vs. ≥ 5 times). Education was categorised to reflect key milestones in the German school system: "<10 years" indicates those who did not complete lower secondary education, "10 years" corresponds to secondary education (Realschule) completion, and ">10 years" includes those with upper secondary or tertiary education. Smoking status was self-reported and categorised as non-smokers (never smoked), former smokers (previously smoked but quit), and current smokers (daily or occasional use). Participants were classified as diabetic if they self-reported a physician-confirmed diagnosis. No biochemical testing (e.g., fasting glucose or HbA1c) was conducted within the study framework. Participants who reported borderline or pre-diabetic conditions without a confirmed diagnosis were categorised as non-diabetic. All covariates were selected a priori based on clinical knowledge. To assess multicollinearity in the regression models, we calculated the variance inflation factor (VIF). All covariates had VIF values well below the accepted threshold of 5, with a mean VIF of 1.27, indicating low multicollinearity and confirming the stability of the estimated coefficients. All statistical analyses used complete case analysis, including only participants with complete data for each model. No imputation methods were used for missing values. Analyses were conducted using Stata/MP 18.0 (StataCorp LLC 2024, College Station, TX, USA). P values <0.05 were considered statistically relevant. The recommendations of the Strengthening the Reporting of Observational Studies in Epidemiology (STROBE) guidelines were applied for reporting (von Elm et al., 2014).

**References**

Jordan, R. A., Bodechtel, C., Hertrampf, K., Hoffmann, T., Kocher, T., Nitschke, I., . . . Micheelis, W. (2014). The Fifth German Oral Health Study (Fünfte Deutsche Mundgesundheitsstudie, DMS V) - rationale, design, and methods. *BMC Oral Health, 14*, 161. doi:10.1186/1472-6831-14-161

von Elm, E., Altman, D. G., Egger, M., Pocock, S. J., Gøtzsche, P. C., & Vandenbroucke, J. P. (2014). The Strengthening the Reporting of Observational Studies in Epidemiology (STROBE) Statement: guidelines for reporting observational studies. *Int J Surg, 12*(12), 1495-1499. doi:10.1016/j.ijsu.2014.07.013

World Health Organization. (1997). *Oral Health Surveys. Basic methods* (4th ed.). Geneva: World Health Organization.

**Suppl. Table 1.** Patient level analyses: Adjusted estimates (β) and their corresponding 95% confidence intervals obtained by regressing mean PD, mean CAL, percentage of sites with PD ≥ 4 mm and percentage of sites with PD ≥ 6 mm from partial-mouth examination on caries status (surface indices).

| **Outcome** | **Adults (35 to 44 years)** | | | | **Seniors (65 to 75 years)** | | | |
| --- | --- | --- | --- | --- | --- | --- | --- | --- |
|  | **Mean PD,**  **β (95% C.I.)** | **Mean CAL,**  **β (95% C.I.)** | **% PD ≥ 4mm,**  **β (95% C.I.)** | **% PD ≥ 6mm,**  **β (95% C.I.)** | **Mean PD,**  **β (95% C.I.)** | **Mean CAL,**  **β (95% C.I.)** | **% PD ≥ 4mm,**  **β (95% C.I.)** | **% PD ≥ 6mm,**  **β (95% C.I.)** |
| **Surface-based indices** | | | | | | | | |
| Healthy surfaces | **-0.007**  **(-0.010; -0.005)** | **-0.013**  **(-0.016; -0.009)** | **-0.215**  **(-0.285; -0.146)** | **-0.031**  **(-0.051; -0.010)** | -0.002  (-0.004; 0.000) | -0.003  (-0.007; 0.001) | -0.32  (-0.093; 0.028) | -0.006  (-0.027; 0.016) |
| DS | **0.029**  **(0.017; 0.041)** | **0.042**  **(0.026; 0.058)** | **0.738**  **(0.400; 1.077)** | **0.139**  **(0.042; 0.236)** | **0.030**  **(0.012; 0.048)** | 0.025  (-0.010; 0.060) | **1.110**  **(0.562; 1.658)** | **0.269**  **(0.071; 0.466)** |
| MS | **0.005**  **(0.002; 0.009)** | **0.015**  **(0.010; 0.019)** | **0.176**  **(0.084; 0.268)** | 0.018  (-0.008; 0.045) | 0.002  (-0.000; 0.004) | **0.014**  **(0.009; 0.018)** | **0.045**  **(-0.018; 0.108)** | 0.016  (-0.006; 0.039) |
| FS | **0.004**  **(0.000; 0.008)** | 0.002  (-0.003; 0.007) | **0.116**  **(0.009; 0.223)** | **0.031**  **(0.001; 0.062)** | -0.000  (-0.007; 0.006) | 0.004  (-0.009; 0.017) | 0.045  (-0.154; 0.245) | 0.034  (-0.038; 0.105) |
| DFS | **0.006**  **(0.003; 0.010)** | **0.006**  **(0.001; 0.011)** | **0.172**  **(0.070; 0.273)** | **0.041**  **(0.012; 0.070)** | 0.003  (-0.003; 0.009) | 0.007  (-0.006; 0.019) | 0.167  (-0.020; 0.355) | 0.061  (-0.007; 0.128) |
| DMFS | **0.007**  **(0.004; 0.009)** | **0.012**  **(0.009; 0.016)** | **0.201**  **(0.128; 0.273)** | **0.033**  **(0.012; 0.054)** | **0.002**  **(0.000; 0.005)** | **0.018**  **(0.013; 0.023)** | **0.079**  **(0.009; 0.149)** | **0.029**  **(0.003; 0.054)** |

PD: probing depth; CAL: clinical attachment loss; % PD ≥ 4mm: Percentage of sites with probing depth greater than or equal to 4 mm; % PD ≥ 6mm: Percentage of sites with probing depth greater than or equal to 6 mm; DS: decayed surfaces; MS: missing surfaces; FS: filled surfaces; DFS: a combination of decayed and filled surfaces; DMFS: a combination of decayed, missing and filled surfaces.

Adjusted for age, sex, region (east/west), education, smoking, diabetes status, toothbrushing frequency, powered toothbrush use, interdental cleaning aids use, periodontal treatment and regular professional oral prophylaxis.

**Suppl. Table 2.** Tooth level analysis: Adjusted estimates (β) and their corresponding 95% confidence intervals obtained by regressing deepest PD and deepest CAL from full-mouth examination on caries status. Mixed-effects models were constructed in the order of tooth type > jaw > patient ID.

| **Outcome** | **Adults (35 to 44 years)** | | **Seniors (65 to 75 years)** | |
| --- | --- | --- | --- | --- |
|  | **Deepest PD,**  **β (95% C.I.)** | **Deepest CAL,**  **β (95% C.I.)** | **Deepest PD,**  **β (95% C.I.)** | **Deepest CAL,**  **β (95% C.I.)** |
| Caries status  (Healthy surface) | **Ref.** | **Ref.** | **Ref.** | **Ref.** |
| D_1-2_ lesions | **0.176**  **(0.020; 0.332)** | **0.305**  **(0.142; 0.468)** | **0.438**  **(0.088; 0.789)** | 0.311  (-0.117; 0.739) |
| D_3-4_ lesions | **0.787**  **(0.509; 1.065)** | **0.758**  **(0.465; 1.051)** | -0.129  (-0.459; 0.201) | **-0.435**  **(-0.861; -0.009)** |
| Filling | **0.469**  **(0.406; 0.532)** | **0.443**  **(0.377; 0.509)** | **0.438**  **(0.339; 0.538)** | **0.383**  **(0.258; 0.508)** |
| Crown | **0.221**  **(0.144; 0.299)** | -n/a- | **0.276**  **(0.197; 0.355)** | -n/a- |
| Tooth type (Incisor) | **Ref.** | **Ref.** | **Ref.** | **Ref.** |
| Canine | **0.092**  **(0.031; 0.152)** | **0.132**  **(0.054; 0.210)** | **0.111**  **(0.020; 0.202)** | -0.074  (-0.267; 0.120) |
| Premolar | **0.268**  **(0.214; 0.323)** | **0.334**  **(0.260; 0.408)** | **0.300**  **(0.210; 0.390)** | 0.157  (-0.048; 0.362) |
| Molar | **0.610**  **(0.555; 0.665)** | **0.718**  **(0.642; 0.793)** | **0.762**  **(0.662; 0.861)** | **1.236**  **(1.001; 1.470)** |

PD: probing depth; CAL: clinical attachment loss; D_1-2_ lesions: caries lesions extending until enamel; D_3-4_lesions: caries lesions extending until dentine.

Mixed-effects models were constructed in the order of tooth type > jaw > patient ID. Models were adjusted for tooth type, age, sex, region (east/west), education, smoking, diabetes status, toothbrushing frequency, powered toothbrush use, interdental cleaning aids use, periodontal treatment and regular professional oral prophylaxis.
